# Neuronal and Vascular Oxidative Stress in Alzheimer’s Disease

**DOI:** 10.2174/157015911798376244

**Published:** 2011-12

**Authors:** Cynthia A Massaad

**Affiliations:** Department of Molecular Physiology and Biophysics, Baylor College of Medicine, Houston, Texas, USA

**Keywords:** Alzheimer’s disease, amyloid β, oxidative stress, reactive oxygen species, blood flow, learning, memory, axonal transport.

## Abstract

The brain is a highly metabolically active organ producing large amounts of reactive oxygen species (ROS). These ROS are kept in check by an elaborate network of antioxidants. Although ROS are necessary for signaling and synaptic plasticity, their uncontrolled levels cause oxidation of essential macromolecules such as membrane lipids, nucleic acids, enzymes and cytoskeletal proteins. Indeed, overproduction of ROS and/or failure of the antioxidant network lead to neuronal oxidative stress, a condition associated with not only aging but also Alzheimer’s disease (AD). However, the specific source of excessive ROS production has not yet been identified. On one hand, amyloid beta (Aβ) has been extensively shown to act as an oxidant molecule. On the other hand, oxidative stress has been shown to precede and exacerbate Aβ pathology. This review will address the involvement of oxidative stress in the context of neuronal as well as vascular dysfunction associated with AD.

## INTRODUCTION

Alzheimer’s disease (AD) is a progressive neurodegenerative disorder affecting 5.3 million individuals in the United States alone [1]. It is characterized by severe cognitive dysfunction and drastic personality changes, mirrored molecularly by two main pathological hallmarks: 1) increased levels of toxic amyloid β (Aβ) oligomers, which eventually accumulate into extracellular senile plaques, and 2) hyperphosphorylation of the microtubule-associated protein tau, which eventually clumps into intracellular neurofibrillary tangles [2,3]. Although the pathological hallmarks of AD are well defined, the characteristics of disease progression vary greatly among individuals, making it difficult to pinpoint specific clinical determinants for the onset and progression of AD. The identification of several gene mutations that predispose the carrier for AD has been instrumental in furthering the current understanding of AD pathology. These include the amyloid precursor protein (APP) and presenilin (PS) genes [4]. Mutations in these genes invariably lead to increased levels of the Aβ protein, the main component of senile plaques, placing this protein at the center of the most popular theory for AD: the Aβ cascade theory also called amyloid hypothesis of AD [2,5]. This theory has influenced and guided AD research for over a decade, and it is still regarded by many as the main hypothesis for pursuing scientific and clinical investigation of the disease. In the past few years, however, many studies emerged and opened way for criticism of the amyloid hypothesis. For example, questions arose when research findings from the amyloid hypothesis did not advance the therapeutic management of the disease, nor did it shed light on the selective affliction of certain individuals. Perhaps, one of them major flaws of the theory is that it does not explain why many aged, but cognitively normal individuals, exhibit abundant Aβ deposition with no signs of clinical AD. Additionally, cases of sporadic AD, with no underlying genetic increase in Aβ, constitute the larger portion of cases. Non-genetic AD has been proposed to be a vascular disorder, whose underlying cause is impaired cerebral blood flow [reviewed in 6]. Although Aβ also contributes to non-genetic cases of AD, it is becoming increasingly clear that multiple factors come into play to precipitate the occurrence of AD.

One such factor, supported by studies from both animal models and human patients, is oxidative stress, which is implicated in AD-related vascular as well as neuronal dysfunction [7-11]. These studies include extensive reports demonstrating the oxidizing potential of Aβ, implying oxidative stress involvement downstream of Aβ [12-25]. However, despite the overwhelming evidence for the pro-oxidant role of Aβ, several studies have demonstrated free radical involvement in AD prior to Aβ-associated cognitive deficits and neuronal pathology and also have implicated ROS increase in modulation of Aβ levels and oligomerization status [26-31].

Although it is clear that oxidative stress contributes to AD, the exact time of this contribution remains ambiguous. This review will first describe oxidative damage, present evidence for its involvement in the etiology of AD, and then will discuss this involvement in the context of neurodegeneration, cognitive deficits, tau hyperphosphorylation and vascular dysfunction associated with AD.

## OXIDATIVE STRESS

Oxidative stress occurs when free radical production exceeds the antioxidant defense mechanisms, leading to oxidative damage of various cellular components, such as proteins, lipids and nucleic acids [10,32-40]. The majority of free radicals within a cell originate in the mitochondrial respiratory chain, where an inefficient electron transfer to oxygen during oxidative phosphorylation and ATP production results in local overproduction of reactive oxygen species (ROS), most notably superoxide anions from complexes I and III of the mitochondrial respiratory chain [41,42]. ROS are necessary for normal cellular functions such as signaling and synaptic plasticity. ROS have been implicated as modulators of memory formation [43-45] and long-term potentiation [46-48], a form of synaptic plasticity widely studied as a cellular substrate for learning and memory [49,50]. They have also been shown to regulate synaptic plasticity-related molecules, including calcium/calmodulin kinase II (CaMKII) [51], the extracellular signal-regulated kinase (ERK) [52-54], and cyclic adenosine monophosphate (cAMP) response element binding protein (CREB) in the hippocampus [53, 55]. Under physiological conditions, ROS levels are maintained at low manageable levels by a cassette of antioxidant molecules such as glutathione and ascorbate, and antioxidant enzymes such as catalase, superoxide dismutase, glutathione reductase and glutathione peroxidase [56,57]. When ROS levels exceed the antioxidant capabilities of the cell, oxidative damage will ensue. ROS are typically categorized as neurotoxic molecules and exert their detrimental effects *via *oxidation of essential molecules such as enzymes and cytoskeletal proteins [35, 58, 59].

High ROS levels are associated with the aging process, where there is ROS overproduction in conjunction with a reduction in the cellular antioxidant defense [60-62]. The brain is particularly vulnerable to oxidative stress because of its high oxygen consumption. Although a relatively small organ by mass, the brain consumes about 20% of the body’s total oxygen due to its metabolic activity and high need for ATP [63]. Neurons are naturally rich in mitochondria to meet this high energy demand, and therefore produce higher levels of ROS [64,65]. The brain is also rich in fatty acids which are prone to oxidative damage [65] and has relatively poor antioxidant systems [64], resulting in limited ability to cope with the large amount of ROS produced. Consistent with the idea of the brain being particularly vulnerable to oxidative stress, excessive ROS have been associated with decreased performance in cognitive tasks, both in physiological aging and pathological conditions that promote oxidative stress, such as AD [27,43-45,66-69]. 

ROS undoubtedly are very important physiological mediators of plasticity and signaling, but can become detrimental to neuronal function when they accumulate excessively in the brain. The following section will present evidence for the involvement of oxidative stress in the events leading to AD.

## ALZHEIMER’S DISEASE AND OXIDATIVE STRESS

In 1986, Martins and colleagues described increased activity of the glucose-6-phosphate dehydrogenase and 6-phosphogluconate dehydrogenase, which are enzymes of the hexose monophosphate pathway, in brains of AD patients compared to age-matched controls [70]. This increase was proposed to be a direct response to the elevated brain peroxide metabolism [70], and represented one of the first reports of the involvement of oxidative stress in AD pathology. Since then, a multitude of reports directly demonstrated extensive oxidative damage in association with AD. This includes lipid peroxidation [32-34, 36], protein carbonylation [10, 37] and oxidative damage to nucleic acids [35, 38, 40, 71]. Lipid peroxidation causes damage at several levels: 1) it generates various reactive aldehydes, which can incorporate into proteins to generate carbonyl derivatives [72], 2) 4-hydroxynonenal (4-HNE), a product of lipid peroxidation can alter the phospholipid asymmetry of the membrane lipid bilayer, leading to apoptotic neuronal loss in both mild cognitive impairment and AD [73] and 3) products of lipid peroxidation can react with mitochondrial enzymes, disrupting mitochondrial energetics, increasing free radical release and therefore leading to further oxidative stress [74]. The addition of carbonyl groups (such as aldehydes or ketones) on proteins occurs as a result of several oxidative reactions. Carbonylated proteins tend to be more hydrophobic and resistant to proteolysis, allowing accumulation of non-fully functional proteins. Oxidative damage to nucleic acids, especially in neurons, inevitably leads to neurodegeneration, which is a characteristic of AD.

Oxidative damage in AD may also be a direct result of oxidative toxicity by Aβ. Indeed, soluble Aβ species have been linked to an increased hydrogen peroxide (H_2_O_2_) and decreased cytochrome C oxidase activity in the Tg2576 mouse model of AD [14]. Aβ has also been shown to induce the formation of mitochondrial ROS *via *activation of NMDA receptors in mature hippocampal neurons [75]. Aβ has also been shown to enter the mitochondria and cause a signaling amplification that inactivates SOD-2 and generates additional free radicals [12]. Additionally, APP the precursor for Aβ, has been shown to be targeted to neuronal mitochondria, with the possibility of contributing to mitochondrial dysfunction and impaired energy metabolism under specific pathological conditions [13]. The production of free radicals by Aβ can also occur following disruption of metal ion homeostasis, including copper (Cu) and iron (Fe) [15,16]; For example, Aβ can bind Cu ions *via *a histidine bridge [17,18], with the possibility of reducing Cu(II) to Cu(I). This reduction reaction releases H_2_O_2_ as a byproduct [19], which causes oxidative damage to cellular components and also reacts with Aβ/Cu complexes to produce the more toxic hydroxyl radical [20]. 

Markers of oxidative stress have also been tightly linked to Aβ: both nucleic and mitochondrial DNA damage have been localized to senile plaques in the brains of AD patients [21]; lipid peroxidation products and damaged membranes are associated with plaques as well [22,23], and advanced glycation end products are present in both tangles and plaques [24,25]. Further involvement of oxidative damage in AD is evident from studies demonstrating elevated levels of antioxidant enzymes in the hippocampus and amygdala of AD patients. The mitochondrial superoxide dismutase (SOD2) has been particularly shown to be elevated in the hippocampus of AD patients [9,76,77]. Incidentally, the increase in SOD2 levels was least pronounced in area CA1 [76] which is required for memory formation and suffers the most neuronal degeneration during AD [78]. Elevated antioxidant enzyme levels indicates a possible compensatory upregulation of antioxidant defenses in response to increased oxidative stress in the brain of AD patients [76].

Importantly, oxidative damage occurring during AD, possibly induced by Aβ, may further exacerbate Aβ toxicity by modulating the amyloid pathway. For example, hydroxyl radicals, in addition to causing cellular oxidative damage to proteins, lipids and nucleic acids, can also react with Aβ causing the formation of dityrosine cross-linking between Aβ peptides which leads to enhanced oligomerization and aggregation [79,80]. Additionally, Siegel and colleagues demonstrated that elevated levels of 4-HNE, a product of lipid peroxidation, promote Aβ aggregation and fibril formation *via *covalent modifications of the protein [81]. In line with these findings, work from our lab recently demonstrated that genetically reducing mitochondrial superoxide by SOD2 overexpression in the Tg2576 mouse model of AD shifts the Aβ pool towards a less amyloidogenic composition and therefore plaque deposition is reduced [27]. Specifically, we showed that, while the total levels of Aβ are unchanged, the ratio of Aβ42 to Aβ40 and also plaque formation are significantly reduced following superoxide reduction. These findings suggest that free radicals of mitochondrial origin interfere with the process of Aβ aggregation and subsequent accumulation into plaques [27]. Along the same lines, Feng and colleagues demonstrated that resveratrol, the antioxidant found in wine polyphenols, interferes with Aβ fibril formation but does not affect the levels of Aβ oligomers [30]. β-secretase, the enzyme responsible for Aβ formation from its precursor APP, has been shown to be upregulated following hypoxia and ischemic injury, suggesting that oxidative stress and hypoxia inducible factor 1α (HIF1α) play a critical role in inducing Aβ overproduction [82]. Conversely, transgenic Tg2576 AD model mice with reduced SOD2 activity, and therefore increased oxidative stress, have been shown to exhibit increased amyloid burden [83,84] and increased tau hyperphosphorylation [84,85], both of which are major hallmarks of AD. Interestingly, the increased tau hyperphosphorylation could be resolved by treatment with the catalytic antioxidant EUK189, indicating that tau pathology is a direct cause of increased oxidative stress [84]. 

While it remains a matter of debate whether oxidative stress or Aβ pathology occurs first in the series of events leading to AD, it is clear that both pathologies coexist and potentiate each other during AD; Aβ is a pro-oxidant and oxidative stress increases Aβ levels. Whether occurring first or subsequent to Aβ increases, oxidative stress is associated with the early stages of AD and is omnipresent during the course of the disease. It contributes to both AD-associated neuronal and vascular pathologies that will be discussed in the following sections.

## NEURONAL OXIDATIVE STRESS IN ALZHEIMER’S DISEASE

### Neurodegeneration

A large number of studies offered ample evidence for the neurotoxic effect of Aβ, however the mechanisms of neurotoxicity remain elusive [reviewed in 86]. Part of the Aβ-induced neurotoxicity has been attributed to the oxidant potential of Aβ, and will be the focus of this section. For example, treatment of rat forebrain neurons with Aβ led to neuronal death that was exacerbated by the presence of Cu and improved with catalase treatment [20,87]. This is consistent with the previously mentioned notion that Aβ/Cu complexes produce H_2_O_2_ [19], indicating that at least part of Aβ toxicity is due to its ROS generating potential. A similar effect was observed in flies, where Aβ toxicity has been shown to be mediated by hydroxyl radicals produced by the Fenton reaction, as manipulations reducing Fe lead to reduced protein carbonylation and prolonged survival of flies, despite the presence of Aβ [88]. These studies provide strong support for AD therapies based on metal chelation to alleviate the pro-oxidant environment created by metals in the brain. This approach, however, was substantially slowed down by the host of side effects of metal chelators and the obstacles encountered in their tissue-specific targeting, a problem that may find its solution in recent nanotechnology advances [89]. Interestingly, Cu has been shown to bind APP and stimulate its processing *via *the non-amyloidogenic pathway, leading to Aβ reduction [90]. Although this may potentially be useful in AD therapeutics, the binding of Cu to APP produces high levels of ROS; the authors suggest that Cu agonists lacking the ROS producing capability should be explored for AD therapy [90]. A more recent study demonstrates that increased levels of APP promote Cu efflux from the cells causing reduced Cu bioavailability and adverse effects on the Cu/Zn superoxide dismutase (SOD1) [91]. This effect is mitigated by dietary intake of Cu which stabilizes SOD1 and reduces Aβ formation [91]. Although this study does not address oxidative stress, the positive effect on SOD1 could possibly have implications on oxidative stress simply by improving the antioxidant capability of the cell. Additionally, the use of EUK-8, a synthetic catalytic free radical scavenger, proved effective against Aβ toxicity in cell culture [92] and supplementation with antioxidants such as blueberries [93], the flavonoids Silibinin [94] and luteolin [95], the polyphenol resveratrol [30,96], Ginkgo biloba extract [31,97,98], epicatechin [99], zeatin [100] and melatonin [101] reduced markers of Aβ-induced oxidative toxicity, such as cell death and accumulation of lipid peroxidation, both *in vitro* and *in vivo*. The antioxidant melatonin prevented the Aβ-induced cell death in organotypic hippocampal slices, and significantly reduced the phosphorylation of tau, glial activation and the Aβ-induced increase of inflammatory cytokines, suggesting that Aβ-induced cell damage is partly mediated *via *oxidative stress-induced neuroinflammation and tau hyperphosphorylation [102]. Glial cells play an important role in the Aβ-induced neurotoxicity leading to dementia. For example, García-Matas demonstrated that Aβ activates astrocytes both *in vitro* and *in vivo* and that this activation is potentiated by the presence of the pro-oxidant agents buthionine sulfoximine, a glutathione synthesis inhibitor, and FeSO_4_, which liberates redox active iron. These agents potentiated Aβ-induced free radical damage, inflammatory damage and apoptosis, suggesting that oxidative stress plays an integral role in Aβ-induced neurotoxicity [103]. This idea is further supported by studies investigating the effects of a novel dietary antioxidant derived from the purple sweet potato anthocyanins (PSPA) on Aβ toxicity *in vitro*. The results of such studies showed that PSPA treatment lead to a reduction in intracellular ROS concomitant with a reduction in Aβ-induced lipid peroxidation and cellular apoptosis, suggesting that Aβ-induced toxicity occurs, at least in part, *via *a pathway involving oxidative damage [104].

A number of studies report the beneficial effects of dietary supplementation with the antioxidant Docosahexaenoic acid (DHA) on cognition and synaptic integrity in various AD models [reviewed in 105]. Hashimoto and colleagues very recently demonstrated that DHA improves the Aβ-induced DNA damage and axodendritic loss and reduces the levels of Aβ-induced lipid peroxidation. The authors suggest that this might be the basis of the DHA-induced amelioration of Aβ-induced neurodegeneration and related cognitive deficits [29]. Similar to DHA, the antioxidant phytochemical cyanidin 3-O-glucopyranoside (Cy-3G) has also been found to reduce cell death elicited by Aβ oligomers in neuronal cell cultures [26]. It also prevented the early events leading to death, such as Aβ oligomer binding to the plasma membrane and disrupting its integrity [26]. These findings further support the early involvement of oxidative stress in Aβ-induced neurotoxicity. Interestingly, the latter two studies [26,29], along with the Ginkgo biloba study [31], also showed that the various antioxidants used inhibited Aβ aggregation and fibrillation, lending support to the notion of oxidative stress involvement in the oligomerization of Aβ into its more toxic form [27,30].

### Learning and Memory

Subjects with established AD-related cognitive dysfunction have been shown to have an imbalance in free radical production and antioxidant quenching mechanisms [9, 10, 106], further implicating oxidative stress in AD-related neuronal dysfunction. As mentioned above, SOD2 deficiency has been shown to exacerbate the AD pathology in mice, as evidenced by increased plaque deposition and worsening of cognitive abilities [83]. It has also been shown to accelerate the onset of a number of behavioral deficits in the hAPP mouse model of AD [107]. Conversely, we and others have shown that overexpression of SOD2 in AD mouse models overexpressing mutant APP (Tg2576 and Tg19959) reduces oxidative stress and prevents memory deficits, as measured by a fear conditioning paradigm and the Morris water maze [27, 28]. These data are in agreement with several studies demonstrating the involvement of mitochondrial free radicals, through SOD-2 deficiency, in increasing AD-related cognitive dysfunction and correlating reduced mitochondrial ROS with improved learning and memory function [83, 84, 107, 108]. Recently, Serrano *et al.,* showed that Aβ-induced ERK phosphorylation in organotypic hippocampal cultures was mediated by redox signaling through NADPH oxidase, suggesting similar mechanisms occur during AD [109]. Because ERK plays a critical role in long term potentiation (LTP) [110,111], which is the cellular substrate for learning and memory [112], this study indirectly implicated ROS produced by NADPH oxidase in the learning and memory dysfunction associated with AD. Further confirmation of the involvement of oxidative stress in AD-related cognitive deficits came from studies analyzing frontal cortex fractions from AD patients, which revealed strong correlations between the levels of synaptic lipid peroxidation, protein oxidation and nitration with the subjects’ cognitive status [113]. The established involvement of oxidative stress in AD-related cognitive dysfunction prompted increased interest in developing antioxidant therapies for the prevention/treatment of cognitive decline during AD. Successful animal studies demonstrated that dietary supplementation with antioxidant molecules or vitamins, including melatonin [101], blueberry extract [114], Ginkgo biloba [115], flavonoids [94,95], carotenoids [100], polyphenols [116], ascorbate [117,118], tocopherol [119] and various combinations of antioxidants [120-124], can decrease oxidative stress and improve memory function, as measured by various spatial and associative memory paradigms. However, the counterpart of such studies in humans yielded conflicting results: While observational epidemiological studies are consistent with the hypothesis that there is an inverse relationship between antioxidant intake and AD risk [125-128], randomized clinical trials do not unanimously support this hypothesis [129-139]. Although this may seem discouraging in terms of antioxidant therapeutics for AD, one should not overlook some important factors that may have affected the results of such trials. For instance, all of the negative trials have in common the lack of information about 1) circulating therapeutic levels of the antioxidant used and 2) whether the antioxidant therapy led to reduced levels of free radicals and/or reduced levels of markers of oxidative stress *in vivo*. This information is important to measure the success outcome of the studies and the lack thereof makes those studies at best inconclusive. Failure of antioxidant therapy in clinical trials may also be due to multiple factors including reduced bioavailability, insufficient length of treatment and/or poor specificity of these treatment regimens, suggesting that an antioxidant therapeutic approach for AD may benefit both from specific targeting and increased antioxidant potency. For example, multiple studies have demonstrated a specific involvement of mitochondrial ROS in AD pathology [27,28,83,84,107], and therefore a specific mitochondrial antioxidant would perhaps show more efficacy than a general antioxidant. A set of synthetic plastoquinone derivatives recently were shown to specifically target the mitochondria and act as potent antioxidants at nanomolar concentrations [140,141]. These rechargeable mitochondrial antioxidants (termed SKQs) have been shown to be effective in terms of decelerating senescence and treating age-related diseases in rodents [140-146]. Although, these agents can become pro-oxidants at higher concentrations [140,141], they still show promise as potent specific antioxidants and therefore, their therapeutic potential should be investigated for AD.

### Tau Hyperphosphorylation and Axonal Transport Deficits

One important function of neurons that is severely affected during AD is axonal transport [147]. Functionality of neurons in the brain is dependent on the cellular machinery that controls anterograde and retrograde transport of a variety of molecules along axons. This machinery primarily involves the microtubule network of the cell, proteins of the dynein and kinesin superfamilies, and several microtubule associated proteins, including tau. The involvement of abnormal tau metabolism in the pathology of AD has been extensively documented [3,148]. Tau is a microtubule stabilizing protein that needs to be partially phosphorylated to function properly. However, when it becomes hyperphosphorylated, not only does it lose its ability to bind microtubules, but it also sequesters normal tau and other microtubule-associated proteins. This leads to the destabilization of microtubules and results in axonal transport deficits [reviewed in 149]. During AD, tau is abnormally hyperphosphorylated [3,148] and therefore it is thought to contribute to the AD-related axonal transport deficits [149]. Aβ and oxidative stress have also been suggested to contribute to the axonal pathology observed in AD [150]. Tau pathology has been shown to occur downstream of Aβ with tau proposed to act as an effector of Aβ toxicity, suggesting that Aβ possibly induces axonal transport deficits *via *tau hyperphosphorylation [151-153]. Furthermore, oxidative stress has amply been shown to lead to increased tau hyperphosphorylation, and therefore it could also possibly contribute to axonal transport deficits *via *a tau mediated mechanism. For example, Melov and associates have shown that increased mitochondrial oxidative stress in AD mice *via *a reduction of the antioxidant enzyme SOD2, leads to exacerbation of the AD phenotype, most notably increased tau hyperphosphorylation at the serine 396 epitope [84]. Recent reports confirm this earlier study and demonstrate that chronic oxidative stress leads to increased phosphorylation of tau at epitopes 399/404 known to occur during tangle formation in AD [85], and that tau pathology and mitochondrial dysfunction exert a synergistic effect during AD and may be responsible for AD-related axonal transport dysfunction [154]. These studies suggest that mitochondrial oxidative stress contributes to tau pathology and could subsequently contribute to axonal transport pathology characteristic of AD. In support of such possibility, recent data from our laboratory specifically demonstrated rescue of AD-related increased tau phosphorylation and reduced axonal transport by overexpression of the mitochondrial antioxidant enzyme SOD2 [155]. 

Interestingly, a recent study described a novel role for the tyrosine kinase Abl related gene (ARG) in phosphorylating tau at tyrosine 394 [156]. Although historically most of the tau phosphorylation sites have been characterized as serines and threonines, recent studies report the possibility of tau phosphorylation at tyrosine residues [157]. This particular study demonstrates that ARG can phosphorylate tau at tyrosine 394 and confirms the occurrence of phosphotau-394 in AD-related tangles [156]. Given the reported role of ARG in oxidative stress and neural development [158], this study strengthens the notion that oxidative stress is directly related to tau hyperphosphorylation.

## VASCULAR OXIDATIVE STRESS IN ALZHEIMER’S DISEASE

There is clearly a prominent role for free radicals in mediating the effects of Aβ on neuronal function [27]. Aβ, however, not only accumulates in neuronal parenchyma, but can also deposit on blood vessel walls in a process referred to as cerebral amyloid angiopathy (CAA) [159,160]. CAA has been documented extensively in AD, with a wealth of evidence linking AD with vascular dysfunction. This evidence includes existence of cerebrovascular disease in the AD brain, blood brain barrier dysfunction, and several common predisposing cerebrovascular risk factors, such as stroke, heart disease, hypertension and atherosclerosis [6, 161-163]. Clearance of Aβ *via *perivascular channels is thought to be impaired during AD, leading to amyloid deposition in blood vessel walls and consequently disruption in vasomotor functions [164-166]. Similar to its effect on neurons, Aβ is also cytotoxic to endothelial cells as well as smooth muscle cells, leading to enhanced predisposition for cerebral hemorrhages [167,168].

The vascular endothelium, which regulates the passage of macromolecules from the circulating blood into tissues, is a major target for oxidative stress and hence plays an important role in transducing the effect of ROS to vascular disease [169,170]. One of the main enzymes of the vascular endothelium is the endothelial nitric oxide synthase (eNOS), which catalyzes the production of nitric oxide (NO) from L-arginine [171]. NO is a signaling molecule that mediates a wide range of effects in different tissues, but most importantly promotes vascular smooth muscle relaxation and hence regulates vascular tone and blood flow [172-174]. Therefore, reduced bioavailability of NO can have serious consequences on vascular function. On the other hand, increased production of NO can also be detrimental, especially in the presence of high levels of free radicals such as superoxide anions. Superoxide reacts with NO much faster than it dismutates [175]. This reaction not only reduces NO bioavailability, but also produces peroxinitrite which is a potent free radical, capable of nitrating tyrosine residues to form nitrotyrosine. In addition, peroxinitrite can be protonated to form peroxinitrous acid, which is a source of hydroxyl radicals, the most potent of the oxygen radicals, with no known detoxifying mechanism for it [176,177]. Evidence on increased nitrotyrosine is well described in astrocytes, neurons as well as blood vessels of AD brains, both in humans and mouse models of AD [11,178,179], indicating a probable involvement of NO dysregulation in the etiology of the disease. Additionally, eNOS levels are increased in brains of AD patients and colocalize with nitrotyrosine [179]. Interestingly, the metabolism of tetrahydrobiopterin (BH4), one of the major cofactors of eNOS, is dysregulated during AD [180,181]. Combined with high levels of superoxide and its derived oxidants, this condition favors the production of NOS-derived superoxide instead of NO [182,183]. This uncoupling effect is mediated primarily by Akt-dependent phosphorylation of eNOS at serine 1177 [184, 185], which alters the kinetics of electron transfer within the enzyme, leading to increased production of superoxide, particularly at low levels of calcium [186]. Although consistent evidence suggests increased NO production during AD, plasma from AD patients actually shows reduced bioavailability of NO. The combination of increased NO production with increased nitrotyrosine immunoreactivity and reduced bioavailability of NO during AD suggests that ROS play a critical role in vascular deficits associated with AD. Specifically, increased free radicals exert a NO scavenging effect, which leads to two deleterious consequences: 1) production of peroxinitrite and ensuing potentiated oxidative stress and 2) reduced bioavilability of NO and ensuing hypoperfusion.

Similar to pharmacologic studies demonstrating the involvement of oxidative stress in AD-related neuronal dysfunction, several groups confirmed oxidative stress involvement in the vascular dysfunction associated with AD. For instance, the flavonoid luteolin has been shown to confer substantial antioxidant protection to the neurovascular unit and subsequently protect from Aβ-induced cognitive dysfunction [95]. Similarly, *in vivo *treatments with the antioxidants N-acetyl-L-cysteine (NAC) and tempol and the peroxisome proliferator-activated receptor γ agonist pioglitazone in AD mice, rescued the cerebrovascular dysfunction and selected markers of AD neuropathology, including cerebral oxidative stress and glial activation [187]. 

Studies with transgenic mouse models support the notion of oxidative stress involvement in vascular function: using mutant mice with reduced expression of SOD2, Wenzel and colleagues demonstrated an increased oxidative stress with disrupted vascular function in these animals in relation to cardiac disease [188]. Iadecola and colleagues used an NADPH oxidase mutant mouse and showed that free radicals derived from the Nox2 subunit of the enzyme mediate neurovascular dysregulation in the aging mouse brain, further confirming the possibility of oxidative stress involvement in AD-related vascular deficits and dementia [189]. In support of these results, we recently demonstrated that overexpression of SOD2 in the Tg2576 AD mice rescues cerebral blood flow impairments characteristic of AD *via *an eNOS mediated fashion [155]. Blood flow deficits in this particular mouse model have been previously reported [190]. 

Collectively, the above-mentioned studies support the involvement of oxidative stress in cerebrovascular dysfunction associated with AD. The mechanism of such involvement has yet to be clarified. In a relatively recent review, Zhu and colleagues propose a nice scheme describing the potential origination of AD in vascular dysfunction, whereby chronic hypoperfusion induces mitochondrial dysfunction leading to oxidative stress, which ultimately leads to impaired cognition and neurodegeneration [191]. Although this hypothesis is very attractive and well supported by published data, it does not shed light on cases of pure vascular dementia or cerebral amyloid angiopathy that fail to progress to AD.

## CONCLUDING REMARKS

The brain is particularly sensitive to oxidative stress because of its high metabolic activity coupled to a naturally lower antioxidant capability. It is therefore likely that oxidative stress plays a critical role leading to neuronal as well as vascular abnormalities in AD. It is also evident that oxidative stress is an early event that plays an important role in the progression of the disease. Although it is unclear whether oxidative stress is initiated by the pathological overproduction of ROS or the decrease in antioxidant capacity, the reality remains that the AD brain lacks the appropriate levels of antioxidants needed to neutralize toxic free radicals. Therefore increasing the levels of brain antioxidants, either as a prophylactic or therapeutic approach, is expected to be beneficial to AD. Lessons from past cross-sectional studies and clinical trials highlight the need for including previously missing information such as circulating antioxidant availability and free radical levels following treatment, and also suggest that AD therapeutics may benefit from the design of potent targeted antioxidants.
